# Successful Long-Term Use of Infliximab in Refractory Pouchitis in an Adolescent

**DOI:** 10.1155/2010/860394

**Published:** 2011-01-12

**Authors:** Jessica Yeates, Mohsin Rashid

**Affiliations:** Division of Gastroenterology and Nutrition, Department of Paediatrics, Faculty of Medicine, Dalhousie University, IWK Health Centre, 5850 University Avenue, Halifax, NS, Canada B3K 6R8

## Abstract

Pouchitis is a common complication that develops after an ileal pouch-anal anastomosis after colectomy for ulcerative colitis. In some cases, pouchitis becomes chronic and refractory to conventional therapies including antibiotics, corticosteroids, immunomodulators, probiotics, and anti-inflammatory drugs. We report a case of an adolescent with chronic pouchitis who not only improved with infliximab therapy but remains in long-term remission with maintenance therapy without any adverse effects. Infliximab is a safe and effective therapy for refractory pouchitis and may obviate the need for pouch removal and a permanent ileostomy.

## 1. Introduction

Approximately one-third of patients with ulcerative colitis will require a colectomy as a result of medically refractory disease, adverse side effects from therapy, or the development of dysplasia [1]. With surgical advancements, most patients who require colectomy are able to avoid a permanent stoma as a result of ileal pouch-anal anastomosis (IPAA). However, up to 60% of patients who undergo IPAA will develop pouchitis, an idiopathic inflammation of the ileal-anal pouch [2]. Pouchitis can present with a variety of symptoms including abdominal pain, diarrhea with or without blood, urgency, fecal incontinence, and weight loss [3]. In many instances, the pouchitis is mild and resolves after medical therapy; however, in 5–10% of cases, the pouchitis becomes chronic and a small subset of these patients develop refractory pouchitis which may require ileal-anal pouch removal and a permanent ileostomy [2]. 

Infliximab (antitumor necrosis factor alpha monoclonal antibody) is an effective therapy for Crohn's disease and acute ulcerative colitis. However, its use in chronic refractory pouchitis has rarely been described. We report a case of successful induction and long-term maintenance of remission of chronic, refractory pouchitis with infliximab in an adolescent.

## 2. Case Report

The patient was diagnosed with severe ulcerative pancolitis at the age of 8 years after presenting with bloody diarrhea, abdominal pain, and weight loss. She was treated with various combinations of corticosteroids, azathioprine, sulfasalazine, and oral mesalamine. Adequate remission could not be maintained, and the patient had several admissions to the hospital over the next two years. After failure of medical therapy, a total colectomy and temporary ileostomy were performed at the age ten years, followed by an IPAA five months later. 

One month following her IPAA, the patient developed abdominal pain, severe diarrhea with urgency and incontinence, and weight loss. A clinical diagnosis of pouchitis was made, and a course of metronidazole was administered. The patient had a very stormy course with severe diarrhea, often with blood, nocturnal stool incontinence, abdominal pain, and weight loss. She was treated with various combinations of metronidazole, ciprofloxacin, mesalamine (systemic and local), steroids (systemic and local), and loperamide. Endoscopy of the pouch showed patchy erythema, and biopsies revealed mild nonspecific chronic and acute inflammation but no granulomas. Another course of metronidazole and ciprofloxacin improved her symptoms, but every attempt to discontinue either of the antibiotics led to an exacerbation in the symptoms. 

**Figure 1:**
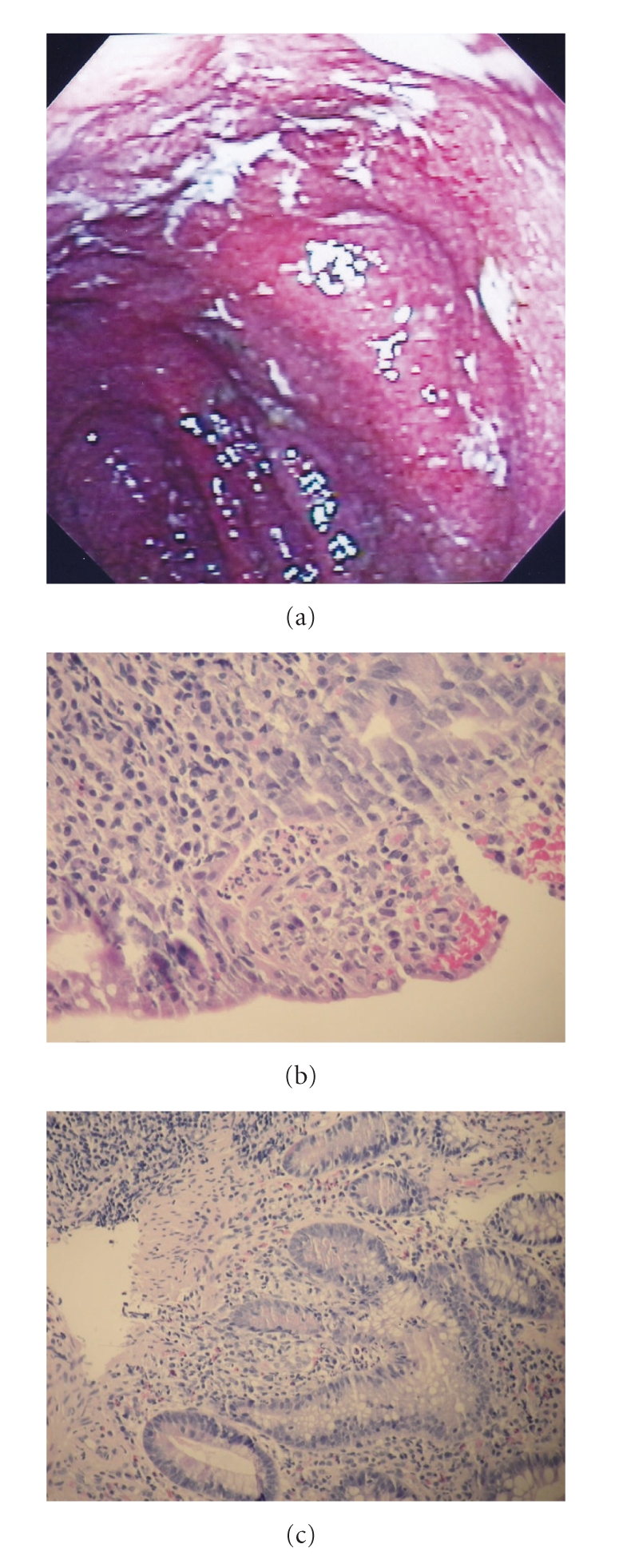
(a) Endoscopic appearance of the ileal pouch before starting infliximab therapy showing diffuse inflammation. (b) Histology of the ileal pouch before starting infliximab therapy showing focal moderate ulceration and cryptitis with a crypt abscess. (c) Histology of the ileal pouch before starting infliximab therapy showing focal chronic regenerative glandular changes.

Another endoscopy a year later showed focal ulcers with surrounding erythema. The histology revealed increase in acute and chronic inflammatory cells, cryptitis, and crypt abscesses. Her symptoms continued to fluctuate despite treatment with various combinations of antibiotics, sulfasalazine, mesalamine enemas, corticosteroids, a probiotic formulation (VSL no. 3), 6-mercaptopurine, and bowel rest with total parenteral nutrition. Another endoscopic examination two years later revealed small ulcerations throughout the pouch and biopsies confirming acute and chronic inflammation. After another severe exacerbation a year later, the pouch was found to be grossly inflamed with diffuse ulcerations (Figure 1(a)). The biopsies showed mild to moderate active chronic inflammation, cryptitis, and crypt abscesses without any granulomas (Figures 1(b) and 1(c)). 

Crohn's disease was carefully excluded after re-evaluation of her history, re-examination of the original proctocolectomy specimen, and examination of the proximal gastrointestinal tract with endoscopy and biopsies. There was no evidence of fistula, clinically or on investigations including barium small bowel study and abdominal computerized tomography. The serology was negative for anti-*Saccharomyces cerevisiae* antibody (ASCA) and positive for perinuclear antineutrophil cytoplasmic antibody (p-ANCA). Stools were repeatedly negative for pathogens including *Clostridium difficile*.

After failure of all medical therapies over seven years, the patient was administered infliximab (5 mg/kg) at 0, 2, and 6 weeks. The 6-mercaptopurine (1.5 mg/kg/day) was continued. The patient had a dramatic response with immediate relief of symptoms and improved weight gain.

**Figure 2:**
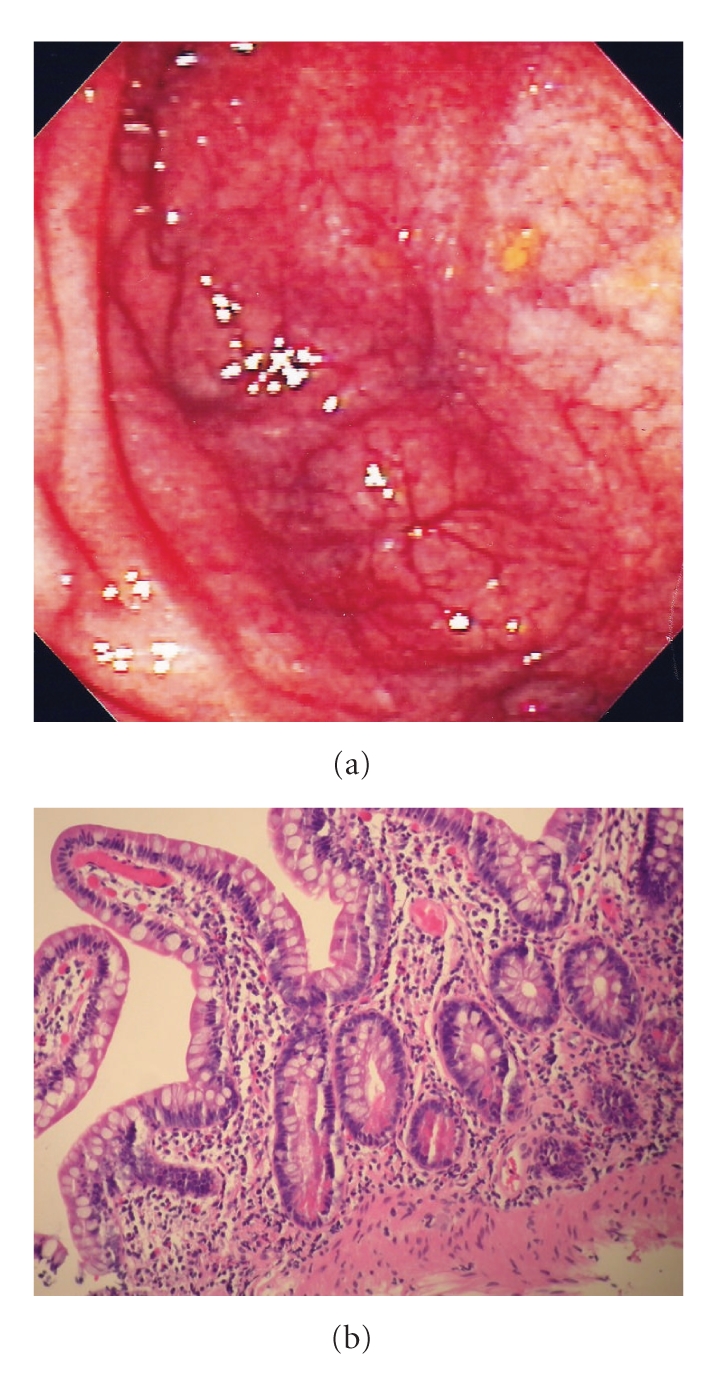
(a) Normal endoscopic appearance of the ileal pouch after infliximab therapy. (b) Normal histology of the ileal pouch after infliximab therapy.

Therapy was continued with infliximab administered every 8 weeks. The 6-mercaptopurine was discontinued a year later. The patient continues to remain in complete remission 36 months after initiating infliximab with regular infusion of 5 mg/kg every 7 weeks. The most recent endoscopy of the pouch (after two years of infliximab therapy) revealed completely normal mucosa with no inflammation (Figures 2(a) and 2(b)). The patient did not experience any adverse effects throughout the treatment with infliximab.

## 3. Discussion

The etiology of pouchitis remains unclear. One hypothesis is that pouchitis occurs as a consequence of dysbiosis of the microbial flora within the pouch; therefore, antibiotics are typically the first-line treatment with the hope of eradicating the dysbiotic flora and restoration of normal flora [4]. Most patients respond to a short course of ciprofloxacin or metronidazole although up to 60% of patients will develop a second episode of pouchitis and 5–10% will require long-term antibiotic treatment [5]. A variety of other therapies have also been tried either alone or in combination including probiotics, budesonide (systemic or local), prednisone, mesalamine (systemic or local), immunomodulators (6-mercaptopurine, azathioprine, and cyclosporine), and octreotide [2, 6–9]. Response to anti-inflammatory and immunomodulator therapies implies that cytokines including tumor necrosis factor-alpha likely play a role in the maintenance of pouchitis.

A report by Molnar et al. recently described the use of infliximab in the treatment of a 16-year-old girl with severe refractory fistulizing pouchitis [10]. Within 4 weeks of total colectomy and IPAA, the patient developed severe fistulizing pouchitis unresponsive to medical therapy. A temporary ileal diversion was performed which resulted in complete symptom relief. However, after reconstruction, severe pouchitis developed six weeks later. Antibiotics, topical budesonide, oral corticosteroid, and azathioprine therapy were all unsuccessful. Within receiving three infusions of infliximab (5 mg/kg), the patient became completely asymptomatic, and remission of her pouchitis was proven by endoscopy and histology. The patient was maintained in remission with regular infusions of infliximab every 8 weeks for 16 months. A fistulizing lesion in a patient in inflammatory bowel disease always raises the question of the underlying disorder being Crohn's disease. As this patient developed fistulizing pouchitis, it is unclear how Crohn's disease was excluded as a possibility in this patient [10].

Viscido et al. studied the use of infliximab in seven adult patients, mean age 32 years (range 19–51), with chronic refractory fistulizing pouchitis [11]. To exclude Crohn's disease, the researchers re-examined the patient's history, proctocolectomy specimen, and small intestine. Infliximab infusions (5 mg/kg) were given to all seven patients at 0, 2, and 6 weeks. Azathioprine was given as a bridge treatment. At 10 weeks, six out of seven patients had a complete clinical response, while the remaining patient had a partial response. Five out of seven patients had complete fistula closure, while the remaining two had partial closure. Patients also experienced complete remission from their extraintestinal manifestations, including erythema nodosum and arthralgia, after the first infusion of infliximab. At final followup, (median 35 months, range 31–57) and with several infusions (mean 4, range 3–7), six out of seven patients were still in complete remission. However, the patient who only had a partial response at the 10-week followup was now in remission, while a patient who had complete clinical remission had recurrence of the pouchitis and fistula. The only adverse effect observed was thoracic herpes simplex infection in one patient which was successfully treated with acyclovir. The investigators concluded that infliximab plus azathioprine is a safe and efficacious treatment of chronic refractory pouchitis complicated by fistulae.

Calabrese et al. did a short-term, single blind, prospective cohort study looking at the use of infliximab in chronic refractory pouchitis complicated by ileitis [12]. Ten patients, mean age 39 years (range 23–56), were treated with infliximab (5 mg/kg) at 0, 2, and 6 weeks. Pouch endoscopy and capsule endoscopic assessment were done at 10 weeks, and clinic assessments at 6, 12, 24, and 48 weeks. Nine out of ten patients had clinical remission, while eight of these patients also developed complete macroscopic remission. One patient without clinical remission did show endoscopic improvement while the last patient did not show any clinical or endoscopic improvement. The same eight patients in remission at 10 weeks were maintained in remission for a minimum of six months (range 8–16 months). There were no significant adverse effects reported.

More recently, a study investigated the use of infliximab in 28 IPAA patients for refractory luminal inflammation (pouchitis and/or prepouch ileitis) and/or pouch fistula [13]. The majority of patients were concomitantly treated with immunomodulatory agents. After ten weeks of starting infliximab, 8 patients with refractory luminal inflammation showed complete clinical response and 3 showed complete fistula response. Of those who responded partially or completely, 56% had a sustained clinical response. Five patients needed a permanent ileostomy. 

Inflammatory bowel disease encompasses two distinct disorders, ulcerative colitis and Crohn's disease. The diagnosis of these disorders is based on clinical, biochemical, radiological, endoscopic, and histological features, as there is no single test to confirm the diagnosis. The development of chronic pouchitis in a patient with ulcerative colitis and IPAA always raises the concern that the underlying disorder could be Crohn's disease. Therefore, it is imperative that Crohn's disease be carefully excluded in such cases. It is possible that some patients reported in the above-mentioned studies had Crohn's disease as the underlying disorder. Infliximab has been used effectively for treatment of Crohn's disease of the ileoanal pouch [14]. Our patient did not have any evidence of Crohn's disease or fistula and is also unique in terms of the longest reported duration of therapy with infliximab in pouchitis in an adolescent.

In conclusion, infliximab is a safe and effective therapy for refractory pouchitis in adolescents who have had colectomy and IPAA for ulcerative colitis. It is also effective in maintaining long-term remission, hence avoiding the need for pouch removal and a permanent ileostomy. A randomized controlled trial is needed to further assess the efficacy of infliximab in the treatment of chronic refractory pouchitis.

##  Conflict of Interests 

The authors declare that there is no conflict of interests.

##  Author's Contribution 

M. Rashid was responsible for conception of the study. Both authors contributed to the design, acquisition of data, analysis and interpretation of data, drafting the manuscript, and final approval of the version submitted.
